# Investigation on Ti6Al4V-V-Cr-Fe-SS316 Multi-layers Metallic Structure Fabricated by Laser 3D Printing

**DOI:** 10.1038/s41598-017-08580-z

**Published:** 2017-08-11

**Authors:** Wei Li, Frank Liou, Joseph Newkirk, Karen M. Brown Taminger, William J. Seufzer

**Affiliations:** 1 Department of Mechanical and Aerospace Engineering, Missouri University of Science and Technology, Rolla, MO 65409 United States; 2 Department of Metallurgical Engineering, Missouri University of Science and Technology, Rolla, MO 65409 United States; 3 NASA Langley Research Center, Hampton, VA 23681 United States

## Abstract

Joining titanium alloy and stainless steel is becoming an urgent need since their outstanding mechanical properties can be utilized integratedly. However, direct fusion joining of Ti6Al4V to SS316 can cause brittle Ti-Fe intermetallics which compromise join bonds’ mechanical properties. In this research, Laser 3D Printing was applied to explore a new Ti6Al4V to SS316 multi-metallic structure. A novel filler transition route was introduced (Ti6Al4V → V → Cr → Fe → SS316) to avoid the Ti-Fe intermetallics. Two experimental cases were performed for comparison to evaluate this novel route’s effect. In the first case, SS316 layer was directly deposited on Ti6Al4V substrate by laser 3D printing, but the sample cracked in the printing process. Then fracture morphology, phase identification, and micro-hardness were analyzed. In the second case, a multi-metallic structure was fabricated via laser 3D printing following the transition route. Microstructure characterization and composition distribution were analyzed via scanning electron microscope(SEM) and energy dispersive spectrometry(EDS). x-ray diffraction(XRD) tests demonstrated the intermetallics were effectively avoided following the transition route. Vickers hardness number(VHN) showed no significant hard brittle phases in the sample. Comparing with directly depositing SS316 on Ti6Al4V, the usage of the novel transition route can eliminate the intermetallics effectively. These research results are good contributions in joining titanium alloy and stainless steel.

## Introduction

Titanium and Ti series alloys have acquired a lot of concerns because they are considered as some of the best engineering materials and biomaterials in the aerospace, nuclear, and chemical industries. These series alloys have excellent mechanical and metallurgical properties such as light weight, high strength-to-weight ratio, and superior heat resistance. In order to combine good mechanical and metallurgical properties of titanium alloys, and either good formability or economic prices of other alloys, there is an upsurge of interest to join Ti alloys with dissimilar structural steels or stainless steels. It is well known that stainless steel is good for weldability and is much more economic than costly Ti alloys, but there is a challenge to join Ti alloys and stainless steels^1^. The traditional heat fusion welding has not yet been technically capable of joining Ti alloy with stainless steel because of a metallurgical incompatibility between them^2^. Direct heat fusion welding of Ti alloy and stainless steel can result in the formation of a variety of intermetallic compounds such as TiFe, TiFe_2_, and so on. These intermetallic compounds are brittle and can embrittle the joint^3^. These brittle formations can reduce the strength of the bound and lead to failure. Cracking at the interface of dissimilar bond is the most common failure type. The above negative factors ultimately result in the risk of fatigue failure during usage and service, even failure may happen in the joining process.

Thus, many previous researchers sought the proper metal or alloy to insert as an interlayer in order to eliminate or relieve the influence of intermetallic compounds^4–7^. The frequently used interlayer metals are Cu^8, 9^, Ni^10–13^, Ag^14^, Al^15^, as well as other more complex alloys^3, 16–18^. The selection of the interlayer material depends on its metallurgical properties with Ti and Fe, especially if the interlayer material can form the intermetallic phase with Ti and Fe in the cooling process after experiencing high-temperature solution annealing. However, the usages of above filler metals unavoidably form intermetallic phases with Ti or Fe in joining Titanium alloy and stainless steel.

**Figure 1:**
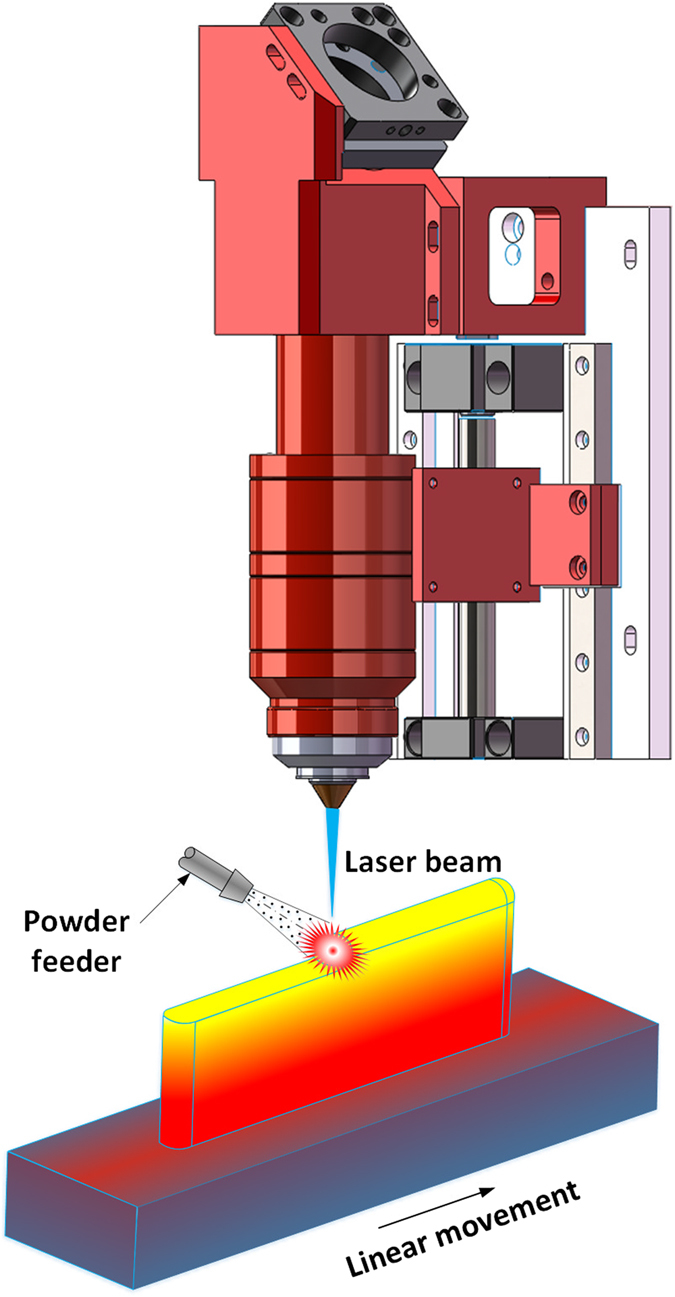
Laser 3D Printing Procedure.

Laser 3D printing is an advanced additive manufacturing technology which can directly produce fully dense, multi-metallic parts. In this study, the laser 3D printing is specialized as laser deposition with blown metal powder. In this process depicted in Fig. 1, a laser beam is used as heat source to melt the metal powder create a melt pool. A powder stream is driven by argon gas flow and continuously conveyed into the melt pool following the powder feeder pipe. The substrate is attached to a three-axis stage, which is driven by computer numerical control system. By moving the substrate according to a desired route pattern, a 2D layer can be deposited, then, a 3D object can be formed through building successive layers on top of one another. With the advantages of high energy density, precise and flexible heating position, and laser beam radius, Laser 3D printing is the most frequently used fusion fabricating method^19^. In addition, laser 3D printing is able to produce a multi-materials part quickly with multi-nozzles by adjusting fed powder types and percentage. The Laser 3D printing process has demonstrated its ability in the area of rapid manufacturing, repairing, remanufacturing, and modification of the metallic components.

Joining Ti6Al4V and SS316 leads to the formation of the large amount of Ti-Fe intermetallic phases. The critical solution was to find an interlayer metal as a transition composition to prevent the formation of an intermetallic phase. However, there is no element that can directly prevent the formation of intermetallic phase with both Ti6Al4V and SS316^17^. Therefore, multi-interlayer metals are necessary to fundamentally address the intermetallic phase and brittleness.

**Figure 2:**
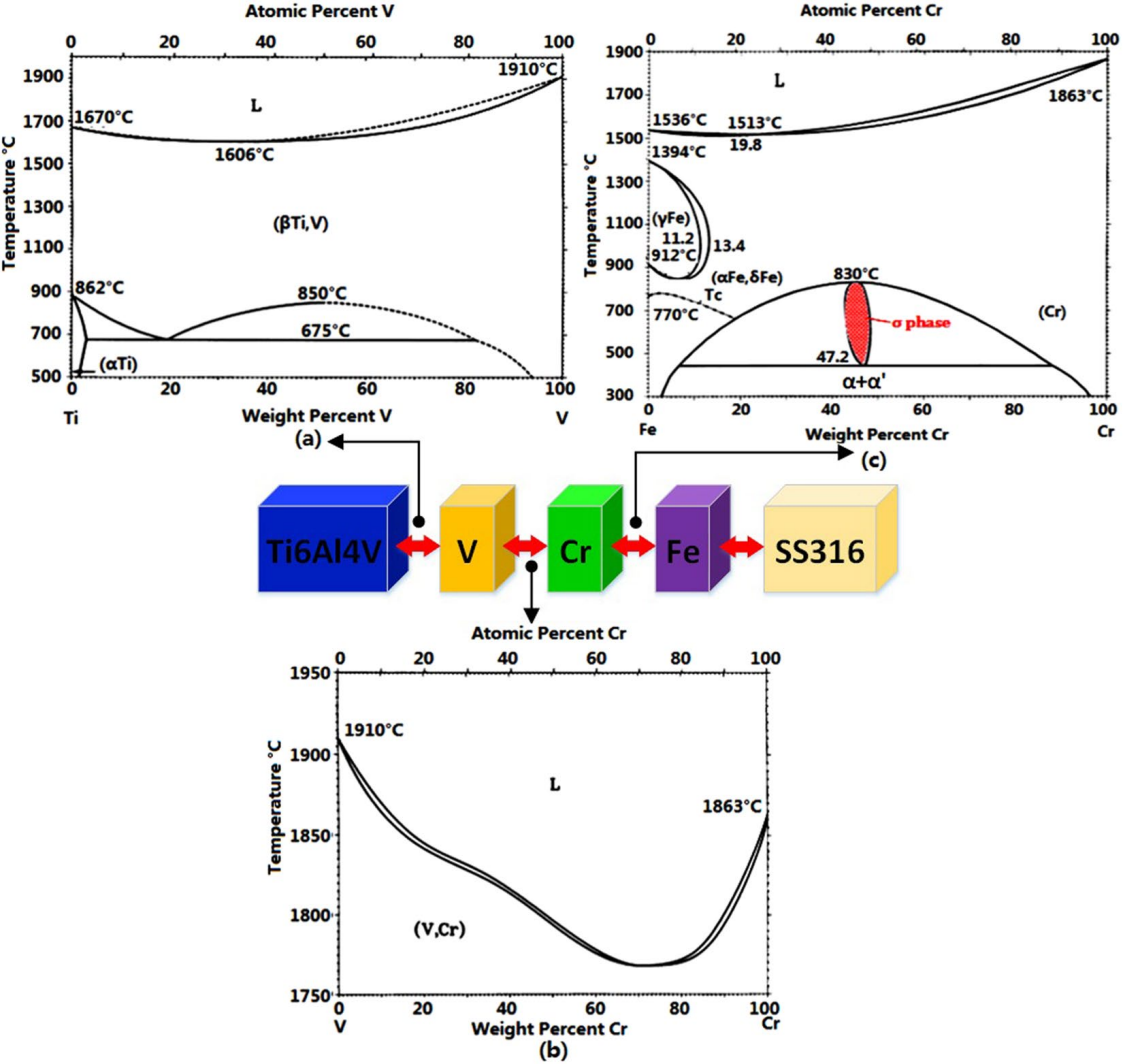
Binary alloy phase diagram of (**a**) Ti-V^32^; (**b**) V-Cr^33^; (**c**) Fe-Cr^34^; and (**d**) composition route.

Vanadium (V) was first considered as a suitable transition metal since V exhibits an excellent ability to form stable solid solution with Ti as shown in the binary alloy phase diagram for the Ti-V system in Fig. 2(a). The beta-phase Ti forms a complete range of solid solutions with V^20, 21^, whereas the behavior of alpha-phase Ti is more limited in this respect. These promising properties of V as a transition metal are further enhanced by thermal expansion coefficients which form a ratio (Ti:V) of 8.5:8.3^17^. As shown in the binary alloy phase diagram for the V-Cr system in Fig. 2(b), V and Chromium (Cr) exhibit unlimited mutual solid solubility across the entire system beneath the solidus, so Cr could be a candidate metal as an adjacent transition composition.

Literature from previous studies^22–24^, has reported a brittle intermetallic sigma phase that is always observed in various series of Duplex stainless steels. The sigma phase often forms under an elevated temperature environment, such as casting, rolling, welding, forging, and aging^25^. In the Fe-Cr binary alloy system, a pure sigma phase exists between 472 °C and 830 °C if the mass percentage of Cr is more than 42.7 wt% and less than approximately 48.2 wt%, as shown in the Fe-Cr binary system in Fig. 2(c). There is obviously no sigma phase formation beneath 472 °C and close to room temperature.

After reviewing previous research and experiment results, the properties of the sigma phase in the Fe-Cr binary alloy system can be summarized as follows. Sigma phase exhibits a tetragonal structure^26^. It is a brittle phase and can decrease the toughness of the system^22^. Sigma phase forms in the cooling process from high temperatures, which is a metaphase in Fe-Cr binary system. To avoid the formation of more than 1% sigma phase, the cooling rate must exceed 0.23 °C/s^26^.

Fe-Cr phase diagram reveals that in the cooling process, if the temperature is lower than around 472 °C, the dominant phases are the α phase and α’ phase. Moreover, the cooling rate is an important factor in controlling the sigma phase’s formation, since large cooling rates can greatly bypass the dangerous temperature range from 472 °C to 830 °C, and reduce the probability of the sigma phase formation. The previous researchers observed the cooling rate in laser 3D printing^27^. The minimum cooling rate value was guaranteed to be larger than 1 °C/s under the laser processing parameters in this study. Based on the above analysis, a new filler transition route was designed: Ti6Al4V → V → Cr → Fe → SS316, as shown in Fig. 2(d).

In this research, to join Ti6Al4V and SS316 by Laser 3D printing, a novel filler transition route was designed to prevent the formation of the intermetallic phase. Two experimental cases were performed to evaluate the effect of this novel route by comparison. SS316 metallic powder was directly deposited on the Ti6Al4V substrate in the first case. The Ti-Fe intermetallic phases formed in this process were investigated through analyzing fracture morphology, phase identification, and vickers hardness test. In the second case, a thin wall sample was fabricated via laser 3D printing following the transition composition route. Then, various material characterizations and analysis were performed to evaluate the new filler transition route. This work sets the basis to fabricate the Ti6Al4V to SS316 multi-metallic structure.

## Results and Discussion

### Directly print SS316 layer on Ti6Al4V substrate

**Figure 3:**
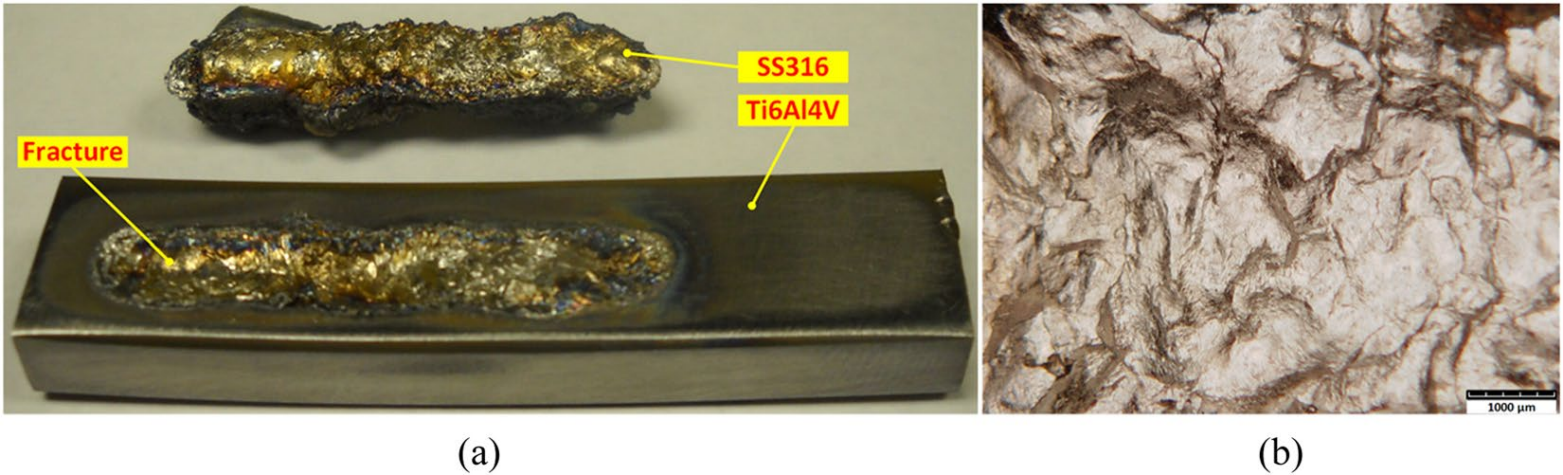
Direct deposition of SS316 on Ti6Al4V substrate. (**a**) SS316 layer fell off from Ti6Al4V substrate; (**b**) Fracture morphology.

In the first experimental case, SS316 metallic powder was directly deposited on the Ti6Al4V substrate by laser 3D printing. In this process, the stainless steel layer fell off from titanium substrate coupled with clear cracking sound (Fig. 3a). The Ti-Fe intermetallic phases formed in this process were investigated through analyzing fracture morphology, phase identification, and Vickers Hardness test.

#### Fracture morphology and phase identification

**Figure 4:**
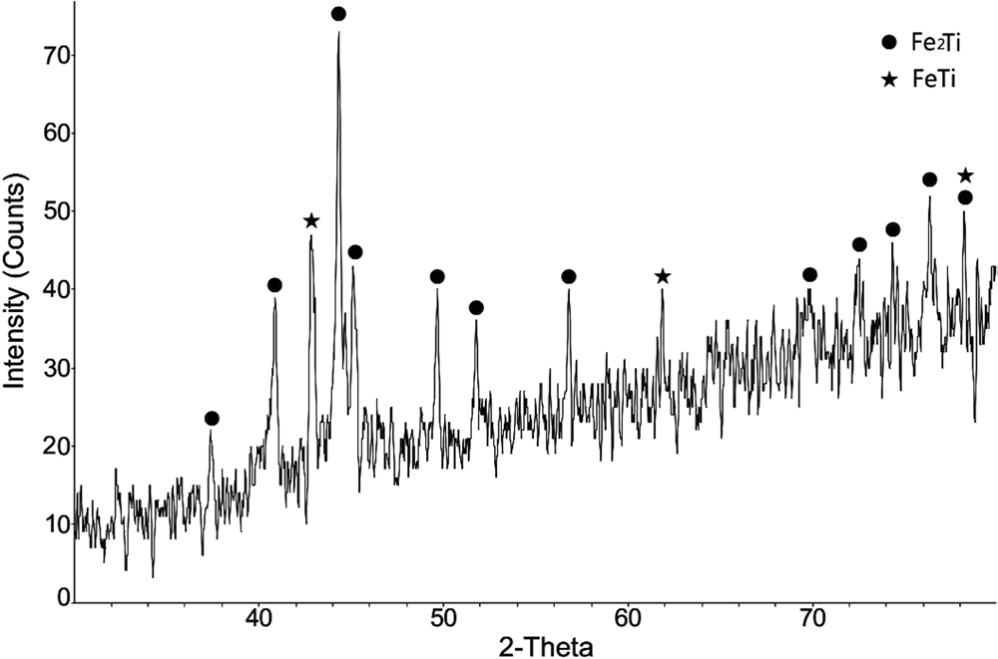
XRD pattern on the fracture area.

It can be clearly observed the fracture morphology is relative smooth, as shown in Fig. 3(b). By observing this fracture morphology, the fracture mechanism is classic cleavage fracture, which is caused by disruption surface’s separating along some crystal plane. Cleavage fracture always happens in body-centered cubic (BCC) and hexagonal close-packed (HCP) metal or alloy. Its crack-evolution is very fast so that resulting in metallic component’s disastrous collapse. This phenomenon indicates that the formed phase in fracture area is very hard and brittle, and almost without any plasticity. XRD test was performed on the fracture area to identify the formed phase. The XRD pattern in Fig. 4 indicates that main intermetallic phases are Fe_2_Ti and FeTi, whose brittleness and hardness caused the direct fracture and clear cracking sound under thermal stress and excessive generation of strains at the interface arising from the thermal expansion difference of titanium and stainless steel alloys.

#### Vickers hardness number distribution on the joint

**Figure 5:**
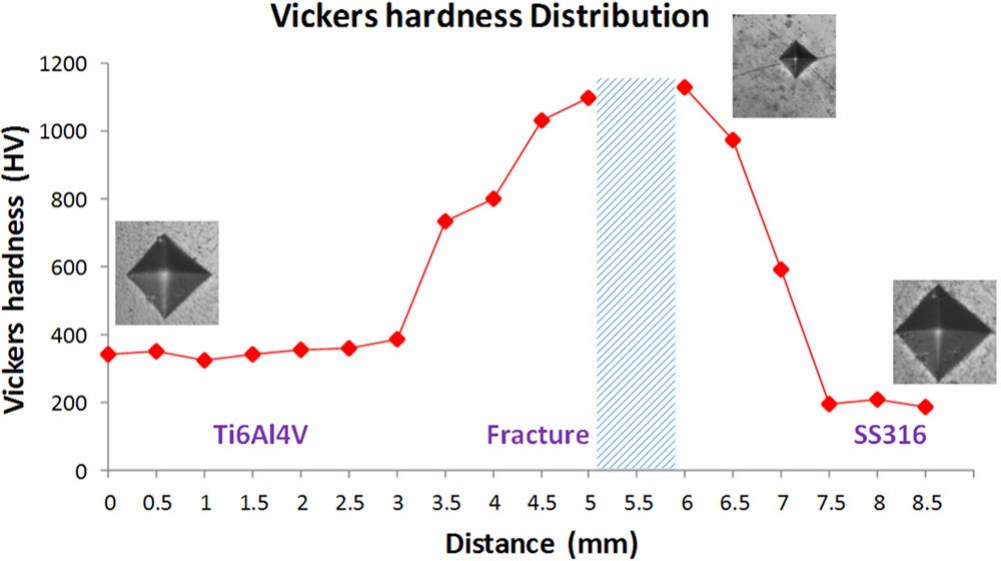
Vickers hardness number distribution along weld joint.

Vickers hardness tests were conducted from Ti6Al4V side to SS316 side, as shown in Fig. 5. The VHN near crack region is much larger than the base alloys on both sides, which demonstrated that the compounds near crack region have poor plasticity. From the VHN distribution in Fig. 5, it turns out that VHN keeps approximately in Ti6Al4V substrate then starts to increase when close to the crack region, and reaches the maximum value of 1130 VHN, where perforative crack happened. The VHN distribution in Fig. 5 illustrates that the formation of intermetallic phases is the primary cause for the failure when directly laser depositing stainless steel powder on titanium alloy substrate.

### Ti6Al4V to SS316 Multi-metallic Structure with novel filler transition route

**Figure 6:**
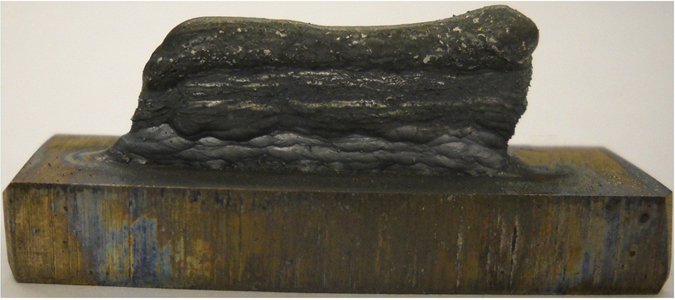
Thin wall multi-metallic sample.

**Figure 7:**
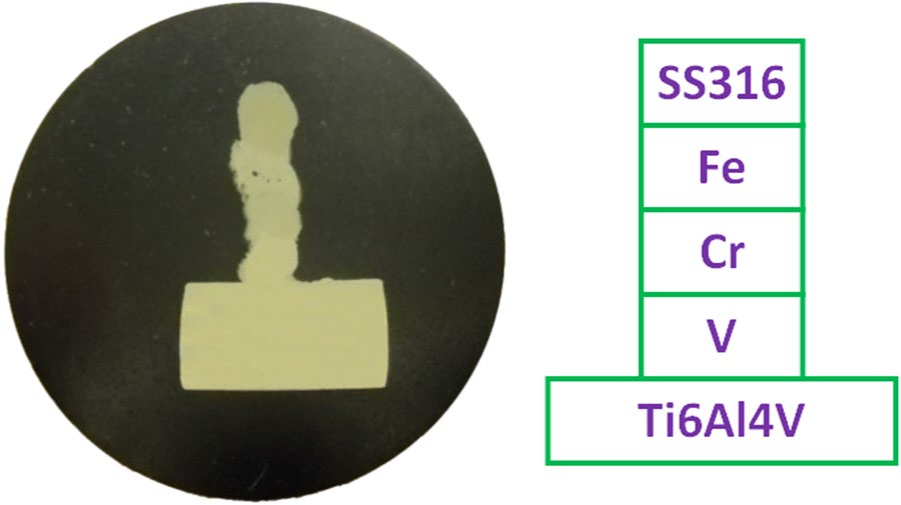
The specimen prepared from cross section of the printed part.

A 3D Ti6Al4V to SS316 Multi-metallic thin wall sample was fabricated layer by layer on the surface of Ti6Al4V substrate by laser 3D printing, as shown in Fig. 6. A specimen (Fig. 7) was cut off along the cross section of thin wall sample for material characterization and tests.

#### EDS and SEM analysis

**Figure 8:**
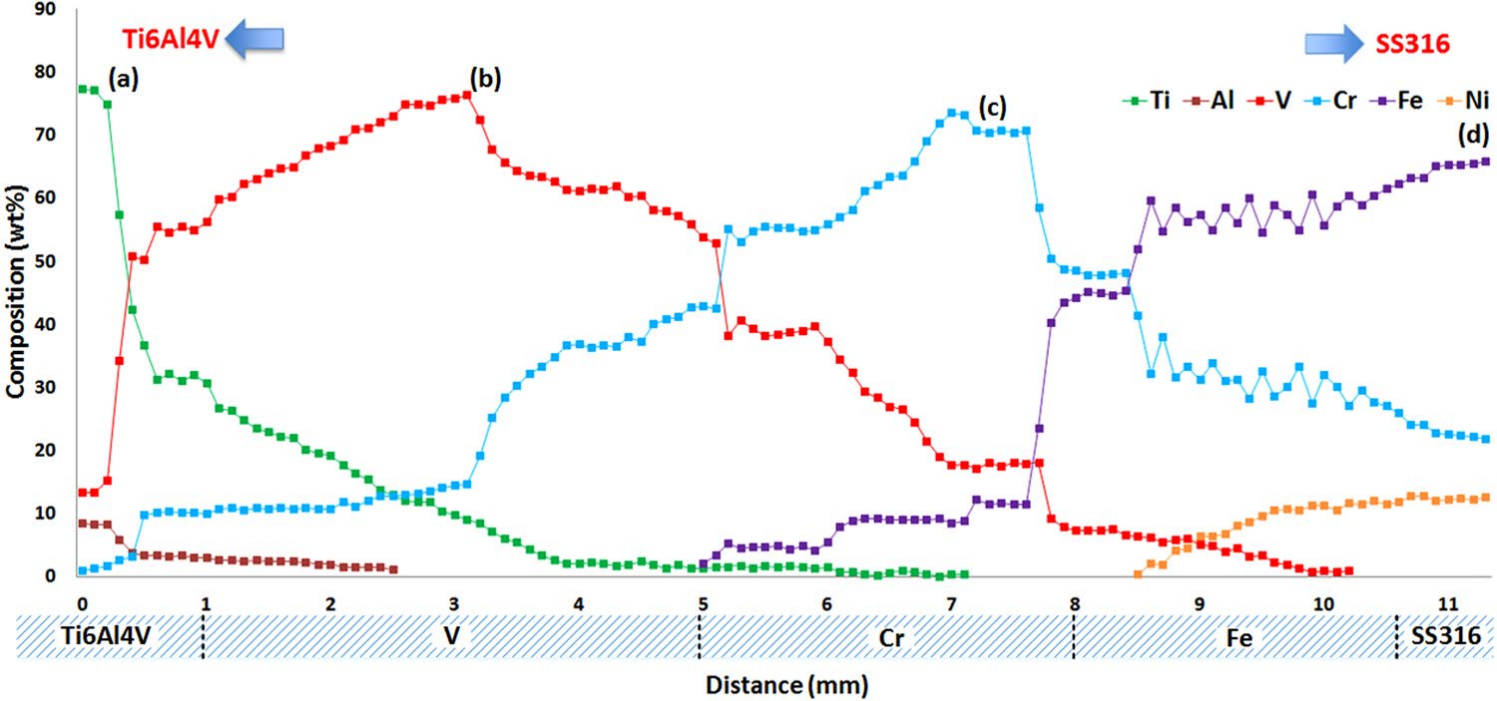
The EDS point test result for composition curve.

EDS was used to analyze the element concentration distribution along the transition composition route. All the EDS point test data for composition curves was plot in Fig. 8. The element distribution curves along the transition composition route on the specimen surface show some interesting features such as intersection, immediate lift and dip, and stagger up and down. These kinds of phenomenon can demonstrate the clear element concentration tendency along the transition composition. Three ridges indicate three transition metals: V, Cr, Fe. V and Cr can diffuse to other metal layers easily. Element diffusion is obvious due to multiple heating and high temperature gradient in laser 3D printing. Another critical phenomenon for mass transfer is the Marangoni convection in the melt pool^28^. The Marangoni force drives the fluid flow near the melt pool surface to flow outward then a convection forms in the melt pool. Due to the convection flow, the bottom material will be lifted upward, on the other hand, the material near the melt pool surface will be transferred downward. The Marangoni convection improves the mass transfer and furthermore improves the diffusion in the multi-metallic structure.

**Figure 9:**
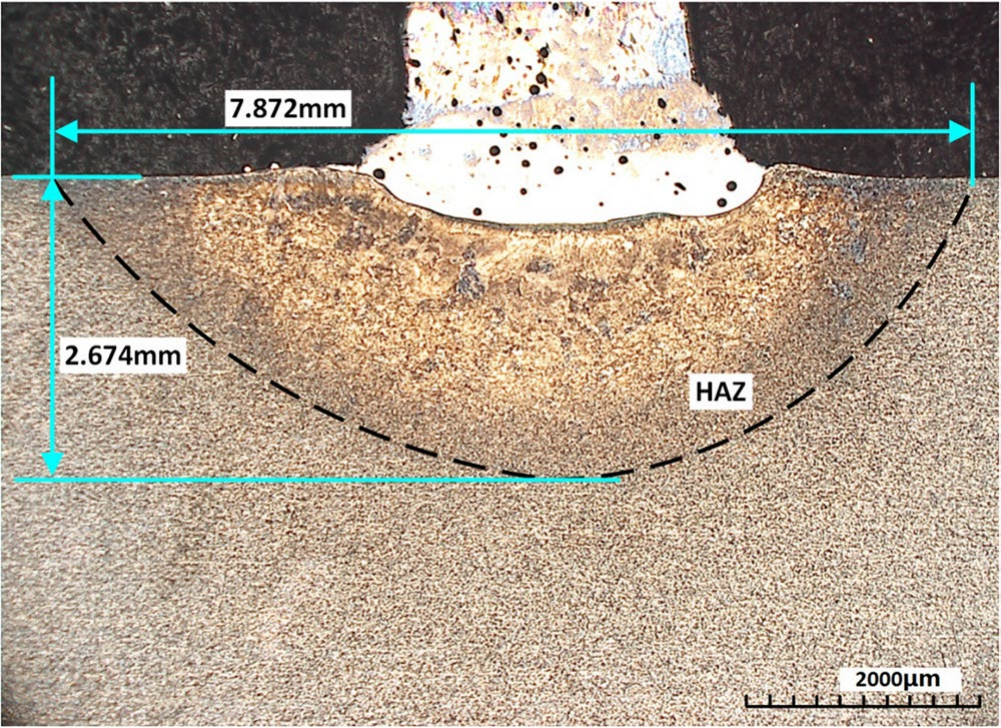
Heat affected zone (HAZ) in the substrate.

**Figure 10:**
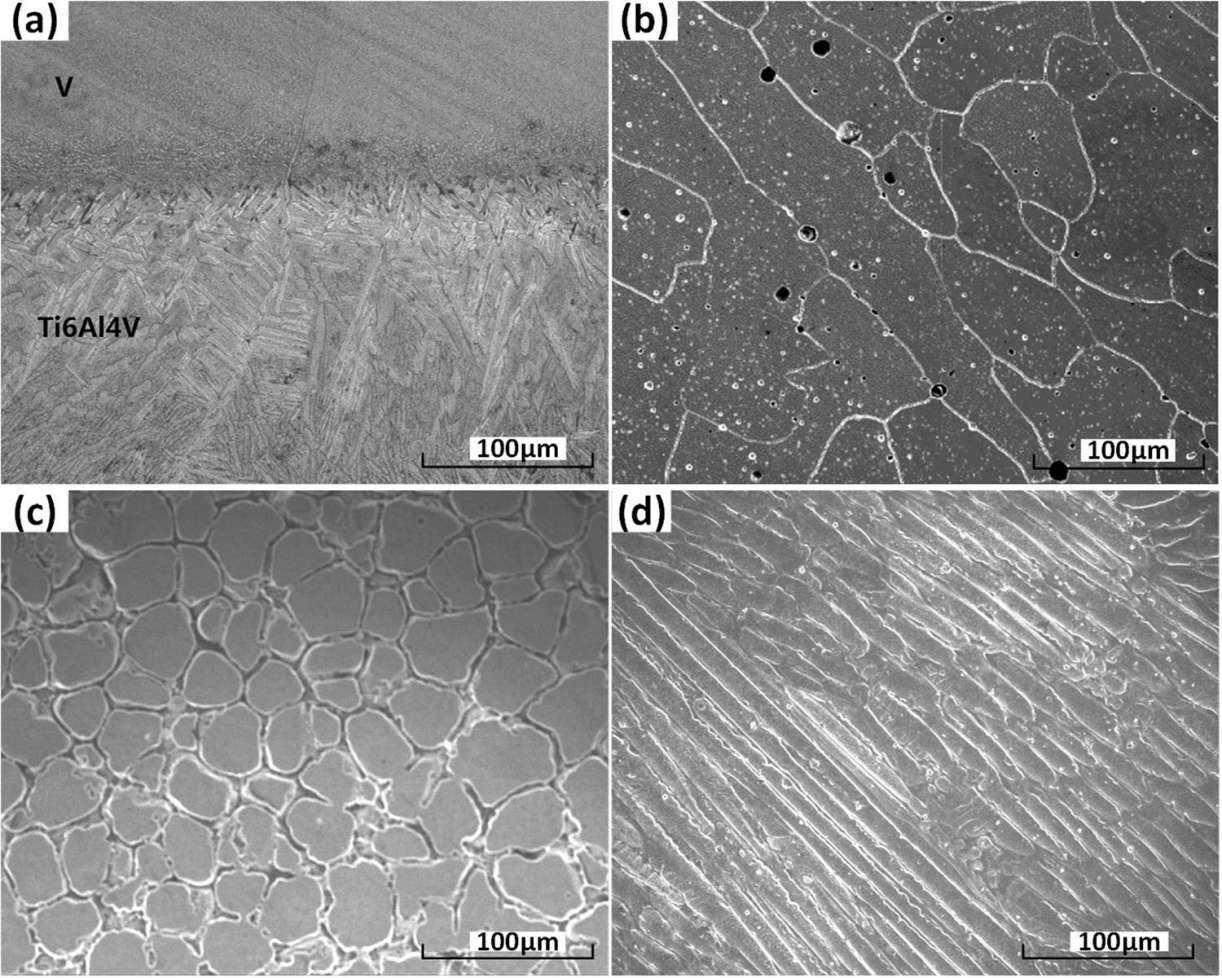
The microscopic images of microstructure at the four sites: (**a**) with maximum wt% of Ti; (**b**) with maximum wt% of V; (**c**) with maximum wt% of Cr; (**d**) with maximum wt% of Fe.

Figure 9 depicted the heat affected zone (HAZ) in the Ti6Al4V substrate, whose depth was 2.674 mm and the width of HAZ was 7.872 mm. To observe the microstructure, SEM tests were done on four sample sites which were selected along the route. The four sites positions were determined by the maximum weight percentage of Ti, V, Cr, Fe, which were indicated with signs (a), (b), (c), and (d) in the Fig. 8. Figure 10 shows the microscopic images of microstructure at the four sample sites.

Figure 10(a) depicts the microstructure close to the Ti6Al4V substrate, where Ti concentration is highest. It is clear to observe the interface between substrate and V layer. On the below side of interface, the Ti6Al4V region exhibits an elongated lamellar-type microstructure. This is caused by the high cooling rate during laser 3D printing process and undergoes rapid solidification. Closer to the interface, thinner and smaller of the microstructure is. From the interface to the Ti6Al4V, it is clear to find the grain’s epitaxial growth in solidification. On the above side of interface, close to V region, some pores are observed which formed in the laser 3D printing process. Figure 10(b) depicts the microstructure with maximum V concentration, which exhibits an equiaxed microstructure. Due to the high cooling rate in rapid solidification, the equiaxed microstructure is elongated approximately along the cooling direction. In the process of laser 3D printing, the powder was carried by argon gas flow then sprayed out into melt pool through powder feeder nozzle. Some gas was dissolved and entrapped in the melt pool, but may not have sufficient time to escape from the melt pool due to rapid solidification in the laser 3D printing. It can be noticed that some spherical pores were observed in V layer. Figure 10(c) depicts the microstructure in the region with highest Cr concentration, which exhibits an equiaxed-type microstructure in this region. Gas porosity is again found in the Cr-rich layer. Figure 10(d) depicts the microstructure where Fe concentration is highest. In this region the microstructure exhibits classic ferrite and austenite grain. High cooling rate in rapid solidification caused the columnar and elongated lamellar-type microstructure.

#### XRD analysis

**Figure 11:**
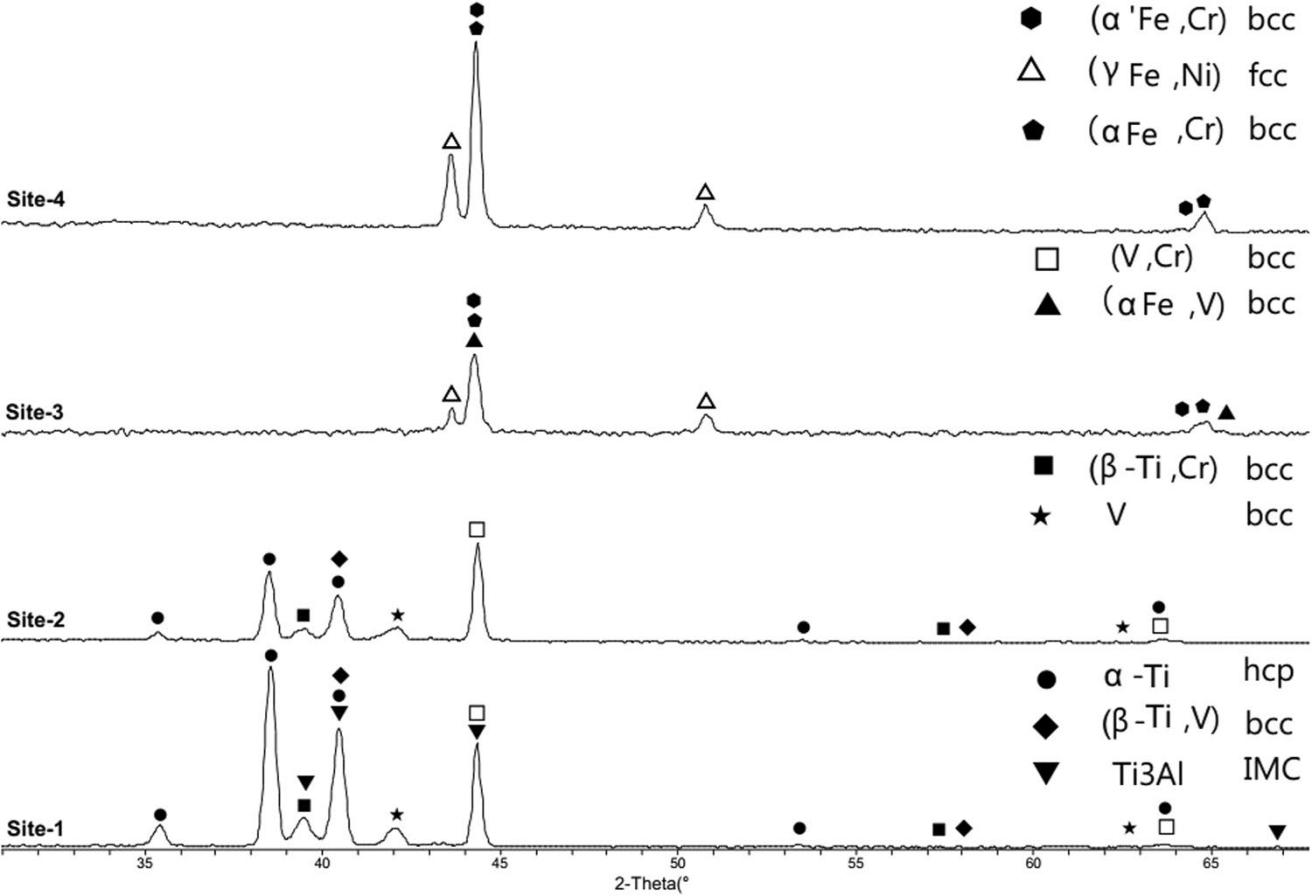
XRD patterns in the four observation sites.

To identify the formed phase in the sample, XRD test was performed on the surface of sample cut from a central cross section of the printed part. Four sites with the maximum weight percentage of Ti, V, Cr, Fe were selected for XRD test. The positions of the four XRD test sites were same with the positions of four SEM test sites in Fig. 10. The XRD patterns are shown in Fig. 11. On the first site, dot is used to represent α-Ti; diamond indicates β-Ti; inverted triangle indicates Ti3Al; all of which above were the primary phases in Ti6Al4V. In addition, (V, Cr) solid solution has strong intensity. Another β-Ti with bcc structure can be detected, which is the solid solution between Ti and Cr. Multiple heating and high temperature gradient in laser 3D printing causes Cr diffuse in V layer and even near Ti6Al4V. Diffused Cr forms into solid solution with V and Ti respectively. Some bcc peaks are lost in XRD pattern, which is mainly caused by preferred orientation. When the specimen is prepared for XRD test, grinding and polishing may cause the multi crystal’s grain directions to be oriented. In addition, in the process of laser 3D printing, metal powders were melted and then re-crystallized. High cooling rate in laser 3D printing results in lathy and tiny grains in the sample. This kind of re-crystallized grain distortion from typical grain structure may cause the missing peaks at least in some specific directions. The XRD pattern on site-2 is similar with site-1. The intensity of α-Ti and β-Ti is weakening, but still obvious. The intensity of (V, Cr) is stronger than site-1, which is basically caused by the higher concentrations of Cr and V on site-2. On the site-3, the α-Ti and β-Ti are not detected, while the intensities of (αFe, V) and (αFe, Cr) keep increasing. (αFe, V) is bcc solid solution of Fe and diffused V. (αFe, Cr) is the solid solution of Fe and Cr, which is also called ferrite with bcc structure. Austenitic fcc solid solution (γFe, Ni) is detected, which may be in SS316 layer, just detected by larger XRD sample area. There are two kinds of Fe-Cr solid solutions α and α’. The (α’Fe, Cr) is high concentration Cr bcc, while (αFe, Cr) is low concentration Cr bcc. Both of them precipitate from the segregation of ferritic solid solution α(δ). In the XRD pattern on site-4, the ferrite bcc has strong intensity, while austenite (γFe, Ni) fcc can also be detected. The phase detected through XRD pattern on site-4 is close to the phase in SS316.

From site-1 to site-3, α-Ti and β-Ti decrease and disappear. On the other hand, ferrite and austenite start to appear from site-3, and increase to the major phases on site-4. Since V, Cr and Fe are added as intermediate metal, some solid solutions are detected in the transition layers. The XRD patterns can verify the material transition design from Ti6Al4V to SS316. In addition, the XRD patterns on the four sites indicate that there is not intermetallic phase.

#### Test for the existence of sigma phase

**Figure 12:**
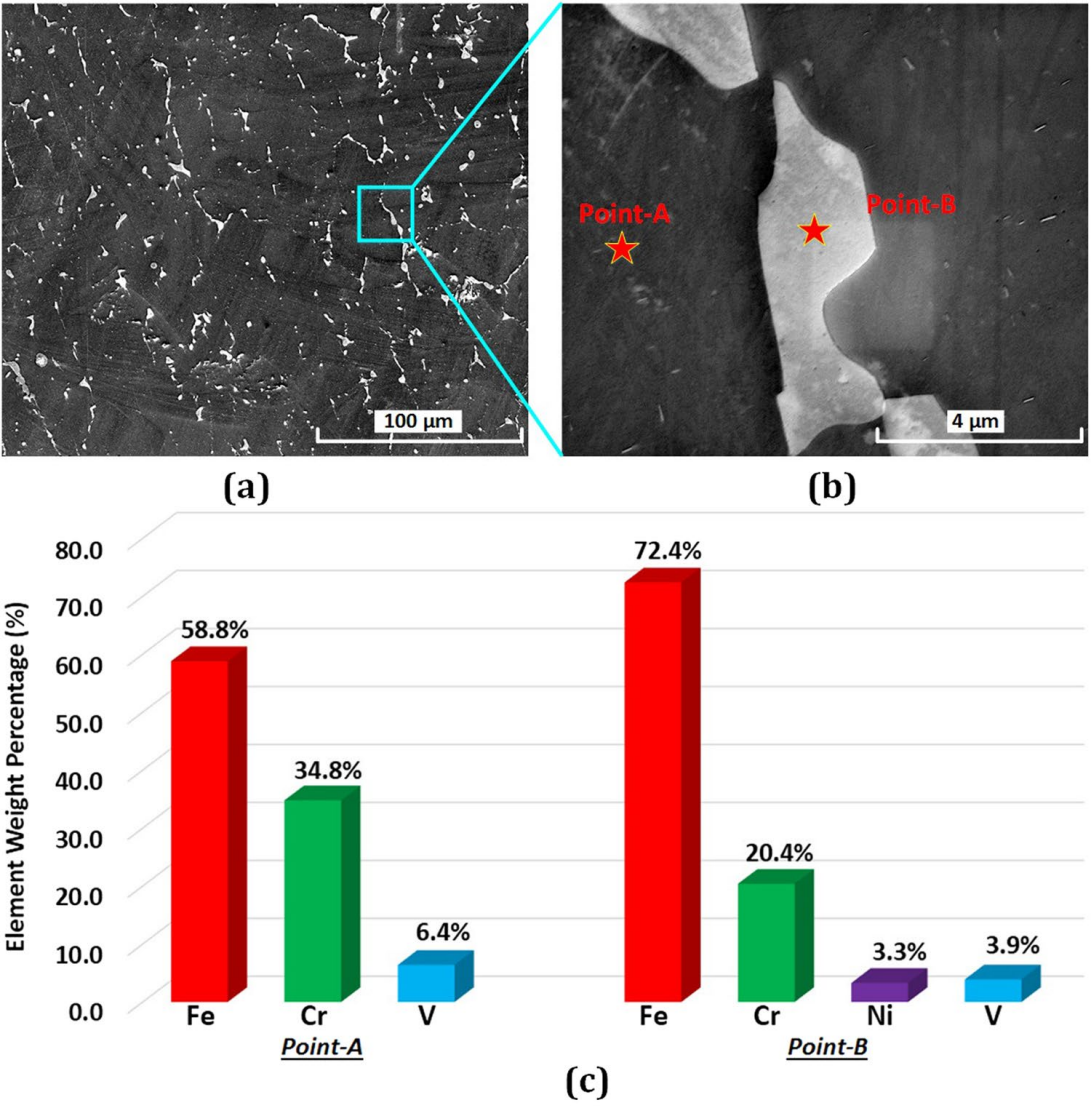
Micrographs at Cr/Fe interface (**a**) and (**b**), and element compositions (**c**).

**Figure 13:**
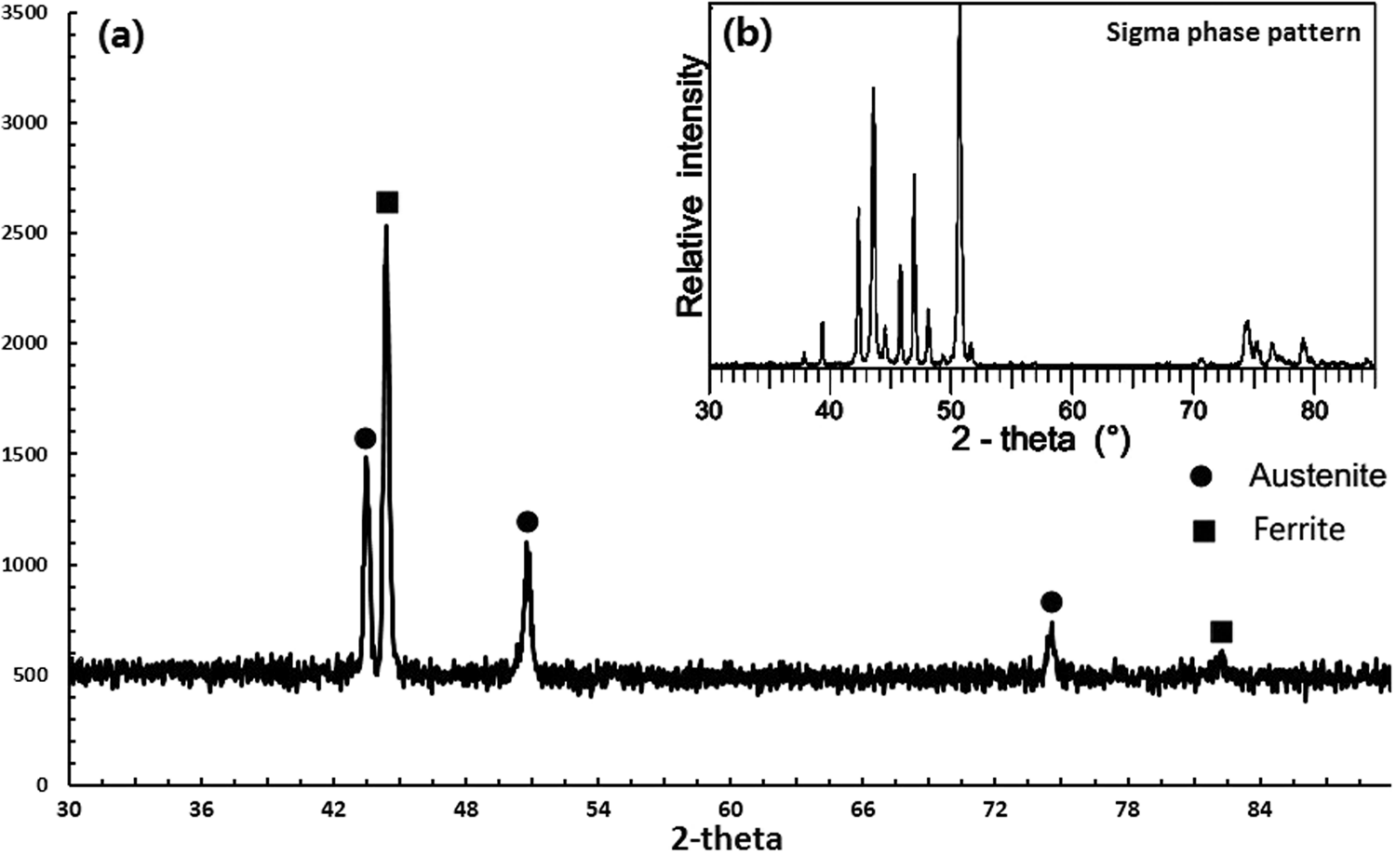
(**a**) XRD pattern at Cr/Fe interface and (**b**) sigma phase XRD patter in the ref. 29.

Referring to the transition route and binary phase diagrams in Fig. 2, sigma phase is only possible intermetallic phase, therefore the test for sigma phase’s existence is necessary. Two SEM images were obtained between Cr layer and Fe layer. As can be seen in these two micrographs (Fig. 12a and b), two phases formed in the solidification process. Light gray phase was embedded in the continuous dark phase. The dark phase is the primary phase at the interface of Cr and Fe. In the micrograph with 12000x magnification, two point EDS tests were done at these two different phases. The element compositions are shown in Fig. 12(c). Two primary metals, Fe and Cr, are detected in the dark phase, whose weight percentages are 58.8% and 34.8 respectively. A small amount of V is another composition in the dark phase. The light gray phase has four metal elements: Fe, Cr, V, and Ni. Fe is the primary element with weight percentage of 72.4%. The Cr composition is less, with the weight percentage of 20.4%. V and Ni weight percentages are less than 5%. Focusing on the region in Fig. 12, one more XRD test was done to detect the primary phase at the interface of Cr and Fe. The XRD pattern is shown in Fig. 13. It is clear to be observed that ferrite and austenite are two phases, in which the ferrite is much more primary. The XRD pattern of sigma phase is also shown in Fig. 13, which was provided by Garin and Mannheim^29^. By comparing the sigma XRD pattern and the detected XRD pattern, there is no formation of sigma phase at the interface of Cr and Fe.

**Figure 14:**
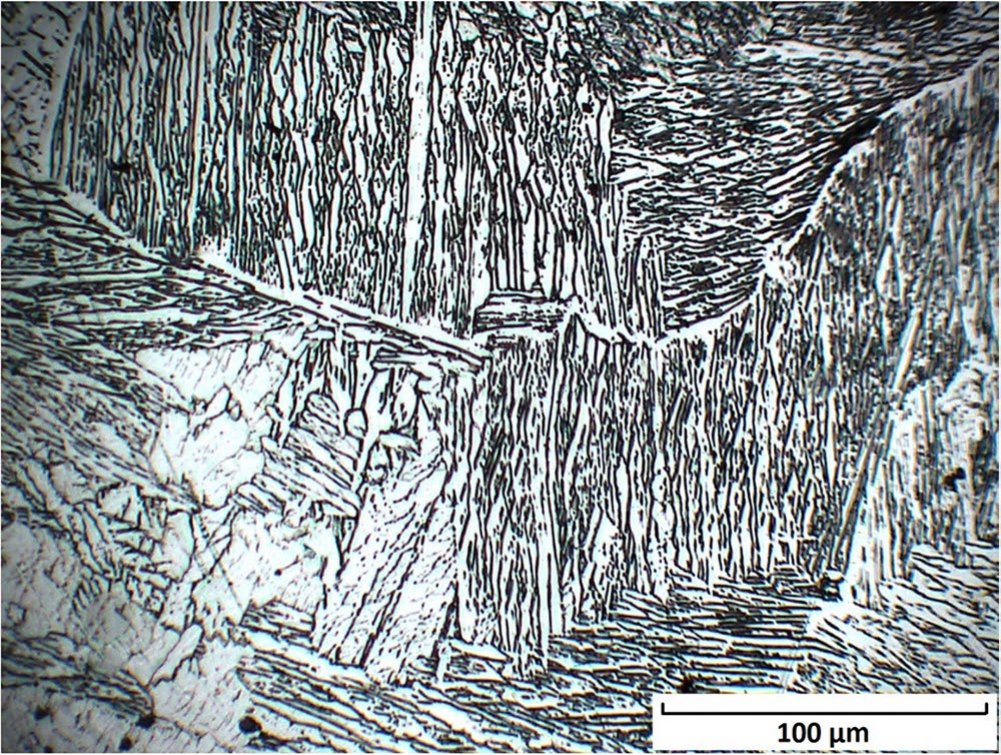
Microstructure morphology at Fe/Cr interface with color etching.

To further detect the sigma phase, the Cr/Fe interface was etched with the reagent (10 g NaOH, 10 g potassium ferricyanide, and 100 ml distilled water), used at room temperature. If the sigma phase formed in the Cr/Fe interface, it was supposed to be colored orange-brown after 60s etched with above reagent^30^. An optical micrograph (Fig. 14) depicted the microstructure morphology in the Cr/Fe interface. An important observation was that, there was not orange-brown region. Long and narrow lathy microstructure was the most primary microstructure in the observation area. Comparing with Lippold and Kotecki’s research^31^, this type of lathy morphology is ferrite microstructure. The lathy morphology forms in the super cooling solidification because of restricted diffusion with high cooling rate. No sigma phase was found with color etching technology.

#### Vickers hardness analysis

**Figure 15:**
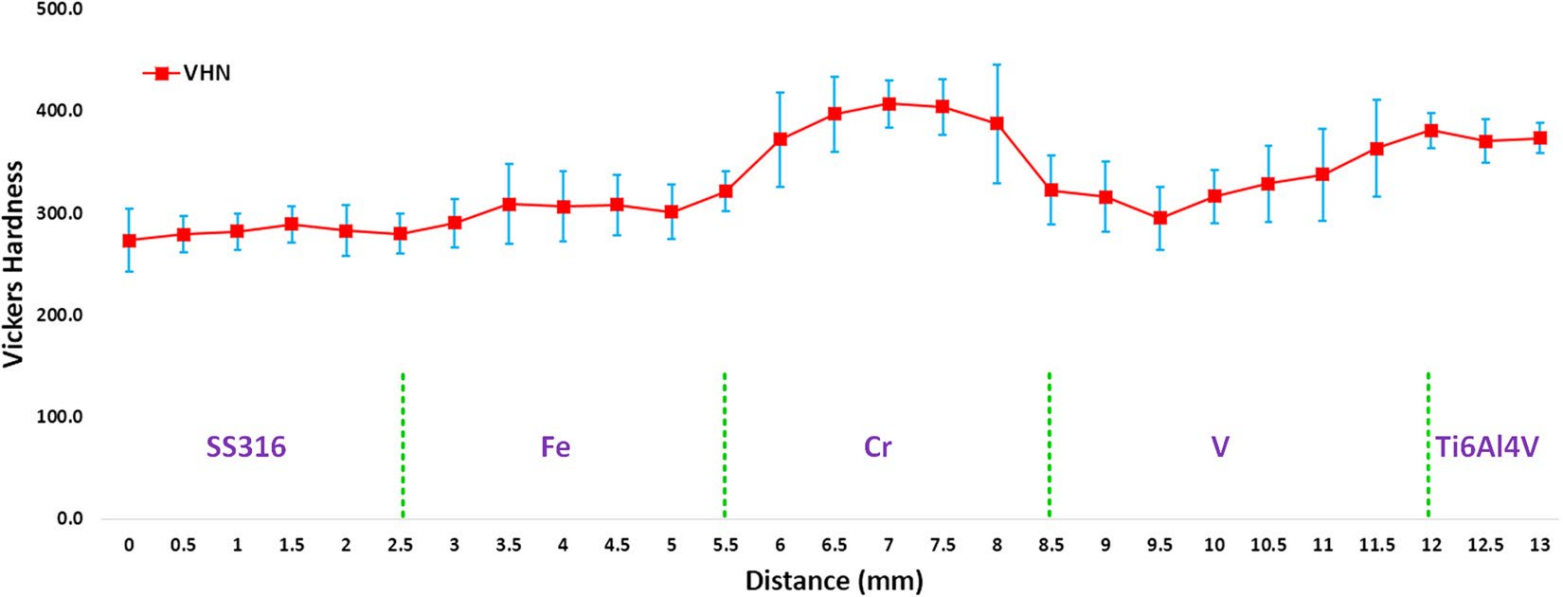
Vickers hardness result along the route.

**Figure 16:**
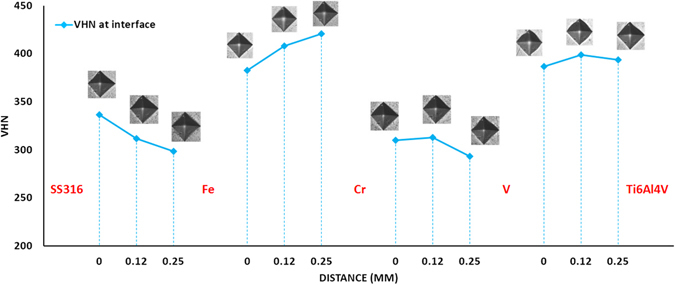
Vickers hardness results at the interfaces.

Figure 15 shows the Vickers hardness test values along with indications of what material region these values belong to. The hardness values were stabilized throughout the SS316 region at 281 ± 19 HV. The highest hardness values were observed around the Fe-Cr interface followed by a slight decrease in hardness value at the Cr-V interface, and then a slight increase in hardness value at the Fe-V interface. The subsequent increase in the Ti6Al4V region was stabilized at 375 ± 16 HV. In the total Vickers hardness number (VHN) distribution, the maximum hardness value was 425.3 HV. There is no obvious area with high VHN in the distribution. All the VHN gradients were slight instead of steep changes. The Vickers hardness result reveals that there is no obvious formation of hard brittle phases in the pure regions of candidate materials or at the interfaces. Figure 16 shows the Vickers hardness value at the interfaces. It can be observed that there is not remarkable high VHN values. All the VHNs are less than 450 at the interfaces.

## Conclusions

The Ti6Al4V/SS316 multi-metallic structure with material route Ti6Al4V → V → Cr → Fe → SS316 was fabricated by laser 3D printing. Some conclusions are generalized as follows:

The multi-metallic structure was fabricated successfully following the material route. The clear element concentration gradient along the transition composition was observed by EDS point tests. The SEM images taken from the sample indicate the elongated lathy microstructure and tiny epitaxial grains. The XRD patterns show that the detected phases exist in form of stable solid solution, and no intermetallic phase was found in the XRD pattern. Further analysis was performed at the interface of Cr/Fe, where the identified phases in XRD pattern were ferrite and austenite phases, but not sigma phase. This conclusion was also supported by color etching technique with light metallography. In the total VHN distribution, there is no obvious area with high VHN. The novel transition composition route design can be used to prevent the generation of intermetallic phases between Ti6Al4V and SS316 alloys.

## Methods

### Materials preparation

Materials used in this experiment were Ti6Al4V, SS316, V, Cr, and Fe. Ti6Al4V and SS316 were regarded as the target materials to be joined together. V, Cr, and Fe were used as filler composition that transitions from Ti6Al4V to SS316 successively. The chemical compositions of Ti6Al4V and SS316 are given in Table 1.

**Table 1:** Chemical composition of the target materials (wt%).

Materials	C	Mn	Si	P	H	S	Cr	Al	V	Mo	O	Ni	N	Fe	Ti
Ti6Al4V	0.08	—	—	—	0.025	—	—	6.76	4.5	—	0.2	—	—	0.25	Balance
SS316	0.03	2	0.75	0.045	—	0.03	18	—	—	3	—	14	0.1	Balance	—

**Figure 17:**
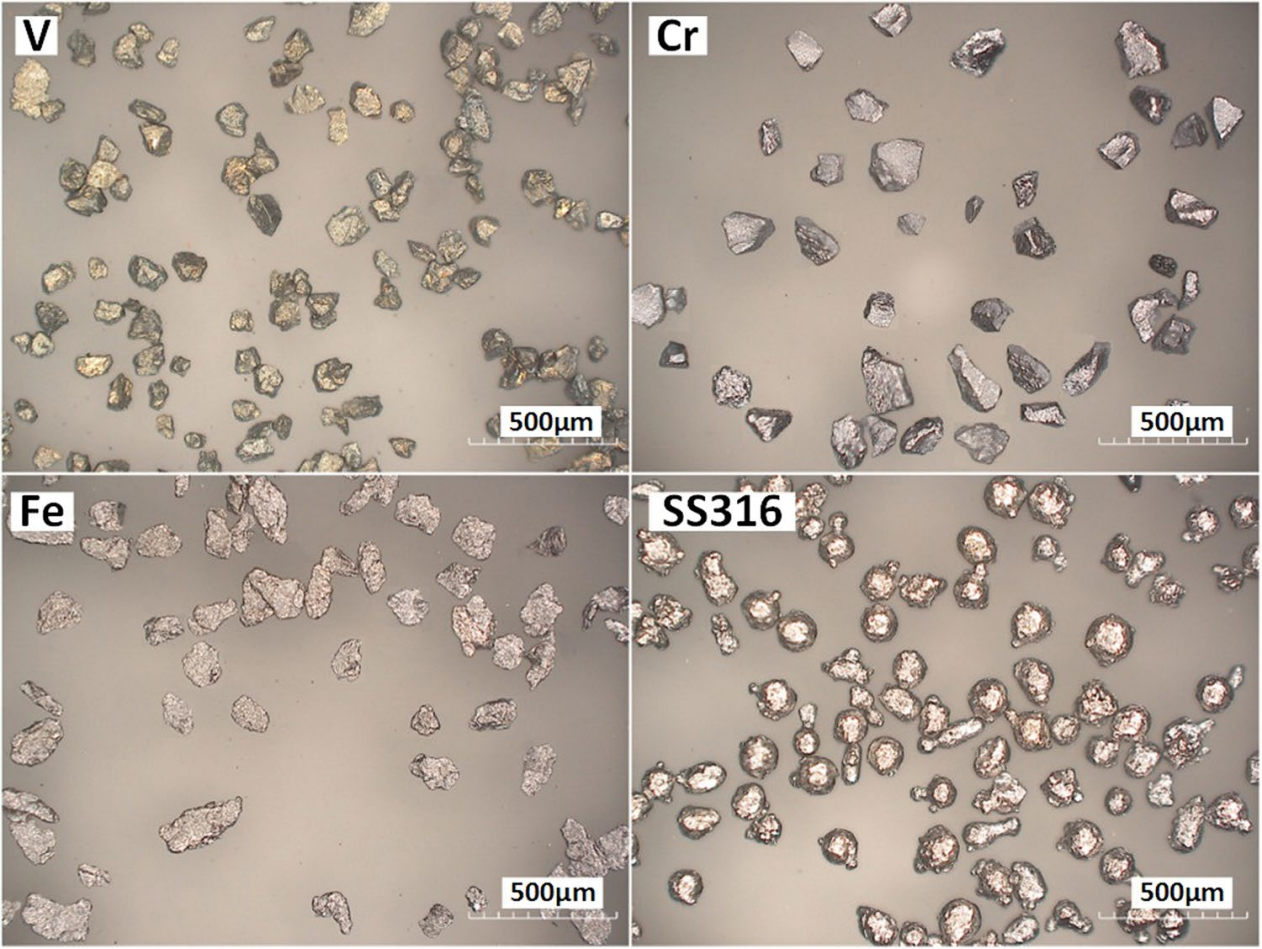
Optical Micrograph of the four types of powder.

The V, Cr, Fe, and SS316 are in the form of pure powder. The powder supplier is Atlantic Equipment Engineers. These powders were characterized to analyze particle shape and size distribution. Microscope images were taken by optical microscope (HIROX Digital Microscope KH-8700). The particles size distributions for all the four types of powder were displayed by the sieve analysis in Table 2. Figure 17 shows four optical micrographs acquired from the four types of powder. The V particles present irregular shape. The Cr particles present a very angular shape. The Fe particles have irregular shapes. SS316 powder particles have a mostly spherical shape when compared to the Fe and Cr powder particles.

**Table 2:** Sieve analysis of V, Cr, Fe, and SS316 powder.

Sieve type	70 mesh	100 mesh	120 mesh	140 mesh	200 mesh	325 mesh
Size (μm)	>212	150–212	125–150	106–125	75–106	45–75
Percentage (%). V	1.3	4.0	8.4	23.7	27.4	35.2
Percentage (%). Cr	0.0	0.6	5.9	9.3	12.7	71.5
Percentage (%). Fe	3.1	9.2	22.4	35.1	21.8	8.4
Percentage (%). SS316	0.0	0.3	3.8	9.0	48.2	38.7

The Ti6Al4V is prepared in the form of a 2 × 0.5 × 0.25 inch bar. In the process of laser 3D printing, the Ti6Al4V bar was used as a substrate. V, Cr, Fe, and SS316 powder were deposited on the top surface of the Ti6Al4V bar.

### Directly print SS316 layer on Ti6Al4V substrate

Laser 3D printing to join titanium alloy and stainless steel is hindered by the formation of interfacial intermetallics caused by metallurgical reactions. Ti-Fe intermetallics are the main obstructive. To investigate the Ti-Fe intermetallics in the process, SS316 metallic powder was printed on Ti6Al4V substrate directly by fiber laser, then analyzed fracture morphology, phase identification, and Vickers Hardness Number (VHN). The laser 3D printing operating parameters are shown in Table 3.

**Table 3:** The operating parameters in printing SS316 on Ti6Al4V by laser.

Laser	Fiber laser CW
Output power (W)	550
Beam diameter (mm)	2
Scan speed (mm/min)	200
Shielding gas	Argon

### Print Ti6Al4V to SS316 Multi-metallic Structure with novel filler transition route

A 3D thin wall sample was fabricated layer by layer on the surface of a Ti6Al4V substrate. V, Cr, Fe, and SS316 powder were deposited successively. The laser processing parameters are detailed in Table 4. A specimen was cut off from the thin wall sample using the Hansvedt Electric Discharge Machine (EDM). The specimen was then mounted on mounting pressure equipment (Simplimet 1000) using a phenolic resin powder. Then, the offcut cutting surface was grinded using abrasive papers from 180 Silicon Carbide Grit to 1200 Silicon Carbide Grit. After that, the specimen was polished using colloidal silica with a median particle size of 0.05 μm. The prepared specimen is shown in Fig. 7. With this specimen, EDS and SEM tests were performed on Hitachi S-4700 Field Emission Scanning Electron Microscope coupled with an Oxford EDS extension. XRD tests were performed with XPERT Pro-type diffractometer to identify the phases in the sample. The Vickers hardness test was performed at room temperature using a Struers Duramin-5 hardness tester with a press load of 9.81 N and loading time of 10 seconds.

**Table 4:** The laser processing parameters in laser 3D printing.

Parameter	Value			
	V	Cr	Fe	SS316
Maximum laser power (W)	1000	1000	700	550
Beam diameter (mm)	2			
Scan speed (mm/min)	200			
Shielding gas	Argon			
Powder feed rate (g/min)	5.1	6.3	7.6	7.2

**Figure 18:**
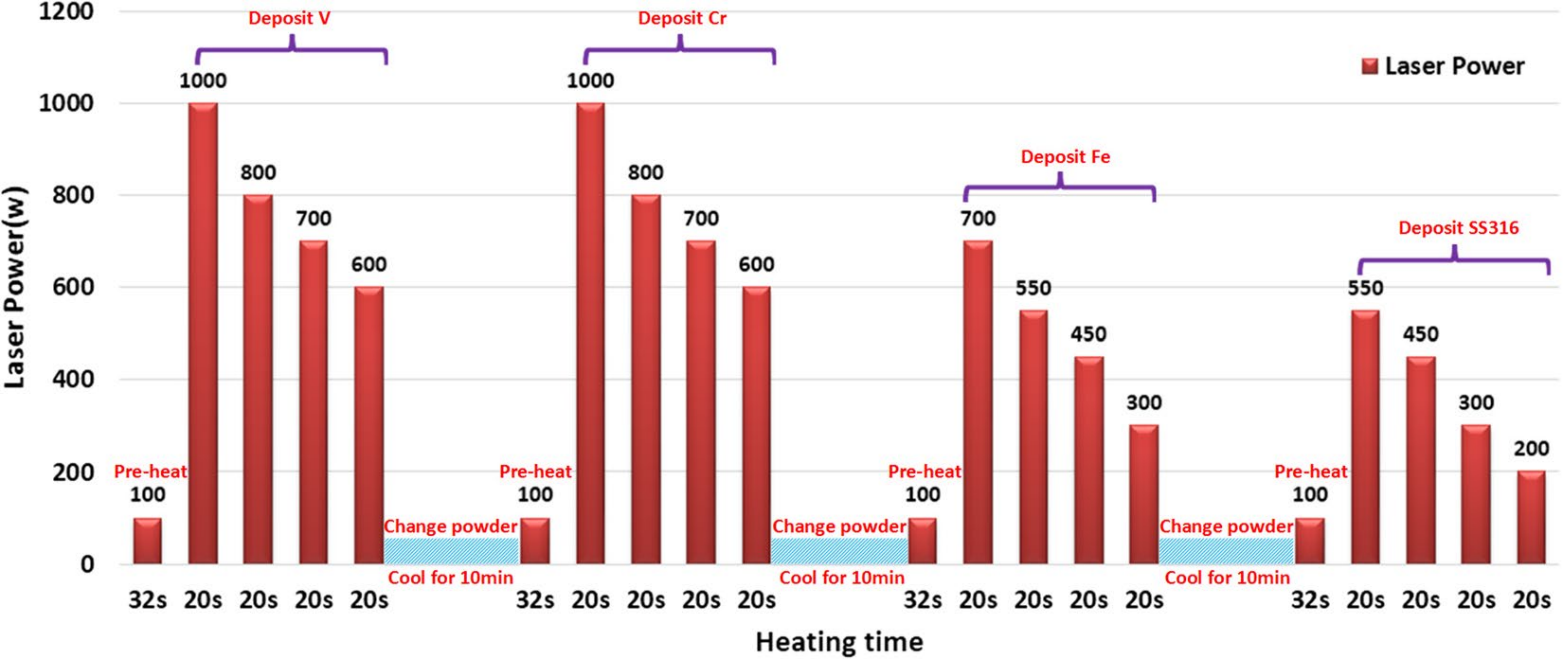
Thermal history in whole 3D printing process.

The printing atmosphere was argon gas atmosphere to avoid oxidization. For each material, 10 layers were printed for each material, with the thickness for each layer about 0.3~0.5 mm. Since melting temperatures for the four metal powders are different (V:1910 °C; Cr:1907 °C; Fe:1538 °C; and SS316:1370 °C), different heat inputs were needed to melt the metal powder to take both efficiency and cost into account. For printing each material, a pre-heating step was designed to heat substrate or previous printed part to form melt pool more quickly. Then a maximum laser power was used to melt the metal powder exiting from powder nozzle. Due to the heat accumulation in the printed part, the maximum laser powder was controlled to decrease gradually. The accumulated heat in the part and decreased heat input can keep the melt pool with approximately stable size, otherwise the melt pool would increase and finally cause the printed part to collapse if keep using the maximum laser power. The maximum laser power for the four metal powders were detailed in Table 4. The printing process was paused for 10 mins to change another metal powder after printing each material. That means the printed part will cool during this powder-changing period. The whole thermal history was depicted in detail in Fig. 18.

Since the four types of powders have different powder morphology, different size distribution, and different densities, the powder feed rates are different under the same argon gas flow rate. The powder flow rates were quantified in Table 4. With the argon gas flow rate of 6 m/s, the powder feed rates for all the powders were consistent over the entire printing process.

The laser 3D printer set-up used in this study consists of the following units: a laser system which provides the heat source, a powder feeding system with a ceramic nozzle, and a linear motor system. (1). an IPG Photonics continuous wave (CW) fiber laser system with a 1.064 μm wavelength was used as the laser heat source. The laser system can provide at most 1000 W laser output power. (2). A commercial powder feeder (Bay State Surface Technologies, Inc, Model-1200) was employed to supply powder in this study. The inert Argon gas was used to carry the powder through the pipeline system, then sprayed through an Al2O3 ceramic nozzle. (3). Three linear motors (AEROTECH, Inc, Model-100SMB2) were employed to generate moving path so that the thin wall sample could be fabricated layer by layer.

### Material characterizations and tests

Microstructure characterization was analyzed via scanning electron microscope(SEM) and optical microscope. Energy dispersive spectrometry (EDS) was used to analyze the element concentration distribution along the transition composition route. 115 sample points uniformly distributed along the route. The bottom of first point is aligned with surface of substrate. The interval between two adjacent points is 0.1 mm. Dwell time for each point is 5 s. X-ray diffraction(XRD) tests detected the formed phase and analyze if the intermetallics formed in the printed sample. The XRD analysis sample was cut from a central cross section of Ti6Al4V/SS316 multi-metallic part. The XRD test was performed on the surface area of cross section, as shown in Fig. 7. Based on the EDS points test results, four sites with the maximum weight percentage of Ti, V, Cr, Fe, were selected for XRD test. The positions of the four XRD test sites were same with the positions of four SEM test sites in Fig. 10. Vickers hardness number(VHN) showed hardness distribution in the sample. All the material characterization and test instruments were detailed in Table 5.

**Table 5:** The instruments used for characterization or tests.

Usage	Instrument	Experiment parameters
SEM&EDS	Hitachi S-4700 Field Emission Scanning Electron Microscope	Magnification: 500x
		Accelerating Voltage: 15000 V
		Emission Current: 8500 a
		Working Distance: 11.9 mm
XRD	PANalytical X’Pert Materials Research Diffractometer	Anode material: Cu;
		Duration time: 30 mins;
		Scan step size: 0.03;
		Scan type: continuous
Vickers hardness	Struers Duramin-5 hardness tester	Load: 9.81 N
		Dwell time: 10 s.
Optical micrograph	HIROX Digital Microscope KH-8700	Magnification: randomly adjust
